# The Lithocatch™ by Boston Scientific: how to use it and how to solve a common problem

**DOI:** 10.1590/S1677-5538.IBJU.2018.0105

**Published:** 2018

**Authors:** Giuseppe Giusti, Marco Lucci Chiarissi, Antonello De Lisa

**Affiliations:** 1 Department of Urology, University of Cagliari, Cagliari, Italy

## Abstract

**Introduction:**

The Lithocatch™ basket is a immobilization device commercialized by Boston Scientific. It allows to collect multiple stone fragments from the ureter. The ability of the basket to capture a large number of stone fragments, is however responsible for a problem connected to its usage: the entrapment of the basket inside the ureter. In this video we explain how to use it and how to solve this problem.

**Material and Methods:**

After positioning the Lithocatch™ over the fragments, the basket is opened and it is rotated through a special handle to collect stones. One frequent problem occurs when too many fragments are collected at once, preventing the extraction of the device. We research our archives to extrapolate the total number of procedures carried out with the Lithocatch™ in the last two years and the total number of complications occurred.

**Results:**

We experienced the above mentioned complication in 16 procedures (14% of the total) of 114 surgeries performed. The way described to solve this complication was efficient and did not produce any damage to the ureter or to the basket.

**Conclusion:**

The Lithocatch™ has an excellent ability to capture small stones so it allows to reduce the length of the procedure. Paying attention to limit the amount of fragments collected, it is possible to avoid the entrapment of the basket. If this complication occurs, the problem can be solved by reducing the size of the stone fragments. The preferable type of energy is the ballistic one.

## ARTICLE INFO

**Available at: http://www.intbrazjurol.com.br/video-section/20180105_Giusti_et_al**

**Int Braz J Urol. 2018; 44 (Video #19): 1262-2**

